# Software-Based Assessment of Well-Aerated Lung at CT for Quantification of Predicted Pulmonary Function in Resected NSCLC

**DOI:** 10.3390/life13010198

**Published:** 2023-01-10

**Authors:** Davide Colombi, Camilla Risoli, Rocco Delfanti, Sara Chiesa, Nicola Morelli, Marcello Petrini, Patrizio Capelli, Cosimo Franco, Emanuele Michieletti

**Affiliations:** 1 Department of Radiological Functions, Radiology Unit, AUSL Piacenza, Via Taverna 49, 29121 Piacenza, Italy; 2 Department of Surgery, General Surgery Unit, AUSL Piacenza, Via Taverna 49, 29121 Piacenza, Italy; 3 Emergency Department, Pulmonology Unit, AUSL Piacenza, Via Taverna 49, 29121 Piacenza, Italy

**Keywords:** non-small-cell lung cancer, pulmonary function tests, computer applications software

## Abstract

Background: To test the agreement between postoperative pulmonary function tests 12 months after surgery (mpo-PFTs) for non-small cell lung cancer (NSCLC) and predicted lung function based on the quantification of well-aerated lung (WAL) at staging CT (sCT). Methods: We included patients with NSCLC who underwent lobectomy or segmentectomy without a history of thoracic radiotherapy or chemotherapy treatment with the availability of PFTs at 12 months follow-up. Postoperative predictive (ppo) lung function was calculated using the resected lobe WAL (the lung volume between −950 and −750 HU) at sCT. The Spearman correlation coefficient (rho) and intraclass correlation coefficient (ICC) were used to the test the agreement between WAL ppo-PFTs and mpo-PFTs. Results: the study included 40 patients (68 years-old, IQR 62–74 years-old; 26/40, 65% males). The WAL ppo-forced expiratory volume in 1 s (FEV1) and the ppo-diffusing capacity of the lung for carbon monoxide (%DLCO) were significantly correlated with corresponding mpo-PFTs (rho = 0.842 and 0.717 respectively; *p* < 0.001). The agreement with the corresponding mpo-PFTs of WAL ppo-FEV1 was excellent (ICC 0.904), while it was good (ICC 0.770) for WAL ppo-%DLCO. Conclusions: WAL ppo-FEV1 and WAL ppo-%DLCO at sCT showed, respectively, excellent and good agreement with corresponding mpo-PFTs measured 12 months after surgery for NSCLC. WAL is an easy parameter obtained by staging CT that can be used to estimate post-resection lung function for patients with borderline pulmonary function undergoing lung surgery.

## 1. Introduction

The main curative treatment of non-small-cell lung cancer (NSCLC) relies on tumor resection [1,2,3,4,5]. Surgery is the preferred treatment for patients with stage I and II NSCLC and for patients with a non-centrally located resectable tumor in the absence of nodal metastasis on both computed tomography (CT) and positron emission tomography (PET) [1]. Nevertheless, the assessment of pulmonary function is required to estimate operative morbidity [1]. The current guidelines provided by the European Respiratory Society (ERS) and the European Society of Thoracic Surgery (ESTS) recommend evaluating the predicted post-operative (ppo) forced expiratory volume in 1 s (FEV1) and diffusing the capacity of the lung for carbon monoxide (%DLCO) in patients with reduced baseline FEV1 and DLCO [6]. The first estimate of residual lung function should be calculated on the basis of segment counting, but in patients with borderline function an imaging-based calculation of residual function is suggested, with encouragement of the use of quantitative CT (QCT) [6]. Emerging techniques, such as CT ventilation imaging, strongly correlate with PET ventilation in evaluating relative lobar function; nevertheless, it requires images acquired at end-expiration and end-inhalation, increasing radiation exposure [7]. QCT has the potential to predict post-surgical lung function by quantifying the ventilated area of the lobe that might be resected [8,9]. Several types of software can calculate, on the basis of CT Hounsfield units (HU) thresholds, the well-aerated lung (WAL) as a surrogate of normal lung [10]. The WAL volume is an easy parameter that can be estimated with both commercial and open-source software by the use of a density mask, even without requiring complete lung segmentation [10,11]. WAL is a predictor of a worse outcome in SARS-CoV-2 disease (COVID-19)-associated pneumonia; nevertheless, its use to predict lung function 12 months after surgery has not been demonstrated yet [11]. Thus, the aim of our study was to test the agreement between postoperative pulmonary function tests (PFTs) 12 months after surgery for NSCLC and predicted lung function based on WAL estimated at staging CT.

## 2. Materials and Methods

### 2.1. Study Population

This retrospective study was approved by the Local Ethics Committee (institutional review board -IRB- approval number 897/2022/OSS*/AUSLPC). Informed consent was obtained from all subjects involved in the study; as stated by the IRB, informed consent was waived when the patient was not reachable in useful time due to organizational reasons. The study included all patients who underwent surgery with curative intent for NSCLC in our hospital between 1st January 2017 and 30th November 2021. The initial sample comprised 189 patients, with a median age of 70 years-old (interquartile range, IQR: 63–76 years-old), 124/189 (66%) males. Exclusion criteria were 1. staging or follow-up CT 12 months after surgery (F-U CT) not performed in our hospital; 2. CT artifacts; 3. thoracic radiotherapy (RT) or chemotherapy (ChT) performed before surgery; 4. surgical pneumonectomy; 5. chronic pleural effusion or pneumothorax at surgery side; and 6. no availability of PFTs 12 months after surgery. The staging process was performed on the basis of the current guidelines of the European Society of Medical Oncology (ESMO) and of the eighth edition of the TNM classification for lung cancer [1,12].

### 2.2. Pulmonary Function Tests

Pulmonary function was evaluated within one month before surgery, while postoperative spirometry was measured at least 12 month after surgery. Spirometry was performed by a flow-sensing spirometer and a body plethysmograph connected to a computer for data analysis (MasterScreen Body/Diffusion, CareFusion, San Diego, CA, USA). FEV1, expressed as an absolute value (liters) and as a percentage of predicted value, was recorded. In addition, %DLCO was also measured by the single-breath method. At least three measures were taken for every variable, with the purpose of guaranteeing the reproducibility of the data.

### 2.3. CT and Computer Analysis

CT scans were obtained with either a 64-row CT scanner (Aquilon; Toshiba, Inc., Tokyo, Japan) or two different 16-row CT scanners (Emotion 16, Siemens AG, Forcheim, Germany; Brilliance 16, Philips Healthsystems, Amsterdam, Netherlands). Patients were scanned on supine position, during inspiratory breath-hold, moving from the apex to the lung bases. The acquisition included an unenhanced scan and single-enhanced phase obtained after 35 s of peripheral intravenous power injection of 90 to 120 mL nonionic contrast material with a 300 mg/mL iodine concentration (Omnipaque 300; GE Healthcare, Little Chalfont, UK) at 3 mL/s, according to patient size. For QCT, only the unenhanced phase was used. Unenhanced CT acquisition was performed with the following parameters: tube voltage range, 110 kV-130 kV; median tube current range, 75–378 mAs; and pitch 1.1; collimation, 1.25 mm. Imaging datasets were reconstructed using sharp kernel (FC 86 for Toshiba scanner, B70 for Siemens scanner, and LungB for Philips scanner) at 1.5–2 mm slice thickness with standard lung window settings (window width, 1500 HU; window center, −500 HU).

All images were anonymized and transferred to a dedicated workstation. A radiologist (D.C.) with seven years of experience quantified WAL by using the commercial software (IntelliSpace Portal; Philips Health System, Best, Netherlands) available in our Hospital. The Chronic Obstructive Pulmonary Disease (COPD) application automatically segmented the whole lung and the airways; later, after applying a noise-reduction algorithm, it was isolated WAL by manually setting the segmented lung included between −950 HU and −750 HU since the best correlation with the visual score had been previously demonstrated [10]. The software output calculated WAL in liters for the whole lung and for each lobe. Additionally, the lung volume lower than −950 HU was recorded as low attenuation areas (LAAs), and the percentage relative to whole-lung volume (%LAAs) was calculated [13].

### 2.4. Surgical Procedures

Indication for surgical treatment was based on the American College of Chest Physicians (ACCP) guidelines [14]. Lobectomy or segmentectomy were performed through video-assisted thoracic surgery (VATS) through one or three port sites (1 cm to 3 cm) without rib spreading under general anesthesia, with single-lung ventilation, using a double-lumen endotracheal tube. We used an endoscopic stapler (Ethicon, Cincinnati, OH, USA; or Covidien, Minneapolis, MN, USA) without reinforcement to divide the fused fissures and excise the pulmonary artery, vein, or bronchus. Hilar and mediastinal adenectomy was performed on the basis of the ACCP guidelines [14]. After anatomic lung resection, a water-seal test was carried out with warm sterile saline under thoracoscopy. A 20F chest tube was placed in the hemithorax before closure of the chest wall. The chest drainage was removed the day after the air leaks disappeared, regardless of the amount of pleural drainage. Patients were sent home after the removal of chest tubes.

### 2.5. Statistical Analysis

Data are reported as median and IQR. Post-operative lung function was calculated by the anatomical segment counting (ASC) method as proposed by Zeiher et al.: ASC ppo-FEV1 (or %DLCO) = preoperative FEV1 (or %DLCO) × [1 − (S × 0.0526)], where S = the number of segments resected; the number of segments for each lobe was identified as follows: right upper lobe (RUL), 3 segments; left upper lobe (LUL), 4 segments; left lower lobe (LLL), 5 segments; right lower lobe (RLL), 5 segments; and right middle lobe (RML), 2 segments [15]. QCT post-operative lung function was estimated by modifying the formula proposed by Sverzellati et al.: WAL ppo-FEV1 (or %DLCO) = preoperative FEV1 (or %DLCO) × [1 − (WAL volume of the lobe to be removed/WAL volume of the entire lung) [9].

Comparisons within measures were determined by the Wilcoxon test. The agreement between measures was assessed by the Bland–Altman method, calculating the Limits of Agreement ± 1.96 SD (LoA), and by the intraclass correlation coefficient (ICC). ICC was interpreted as follows: <0.40, poor agreement; 0.40–0.54, weak agreement; 0.55–0.69, moderate agreement; 0.70–0.84, good agreement; and 0.85–1.00, excellent agreement [16]. Relations between measures were estimated by the Spearman rank correlation coefficient (rho). A *p* value of less 0.05 was considered significant. Statistical analysis was performed using MedCalc software, version 14.8.1 (MedCalc Software, Ostend, Belgium).

## 3. Results

### 3.1. Study Population

**Figure 1:**
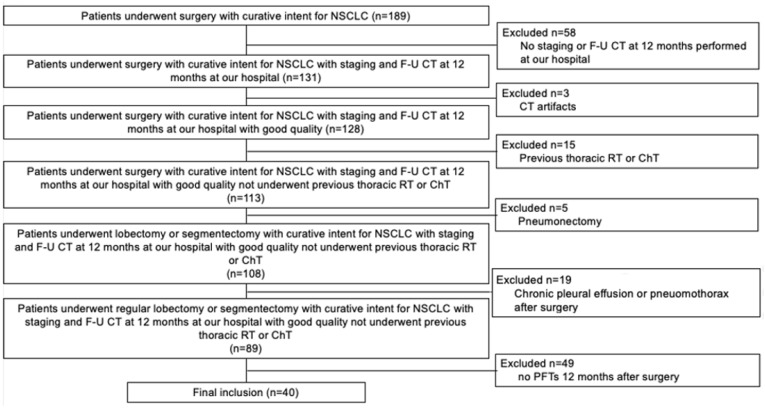
Diagram shows the patient selection process. ChT = chemotherapy; CT = computed tomography; F-U = follow-up; NSCLC = non-small-cell lung cancer; PFTs = pulmonary function tests; and RT = radiotherapy.

From 189 patients initially included, the final cohort included 40 patients with a median age of 68 years-old (IQR 62–74 years-old); 26/40 (65%) patients were males (Figure 1). Lung cancer features, surgical details, and patients demographics are shown in Table 1. The majority of the patients were smokers (35/40, 88%); in particular, 26/40 (65%) were former smokers. COPD was diagnosed in 25/40 (62%) of the patients. The majority of the patients were in stage I, identified in 27/40 (68%) of the patients; in particular, the most frequent tumor stage was IA1 (16/40, 40%). With the exception of 2/40 (5%) patients, all tumors were adenocarcinoma (38/40, 95%). The most frequent location was right upper lobe (18/40, 45%). Surgical segmentectomy was performed in 9/40 (22%) patients, while the remaining patients (31/40, 78%) underwent a standard lobectomy.

### 3.2. Pulmonary Function Tests and Quantitative CT

Median preoperative FEV1 was 2.33 L (IQR, 1.68–2.76 L), which corresponded to a %predicted FEV1 of 85% (IQR 67–101%). The preoperative median %DLCO was 71% (IQR 58–83%). The median time between preoperative and postoperative PFTs was 15 months (IQR 13–16 months). The postoperative median PFTs values were significantly lower than the preoperative values, with FEV1 of 1.94 L (IQR 1.66–2.35 L; *p* = 0.0005), a %predicted FEV1 of 78% (IQR 64–88%; *p* = 0.006), and a %DLCO of 63% (IQR 52–72%; *p* < 0.0001).

The total median WAL at baseline CT was 4.81 L (IQR 4.41–5.38 L), divided by lobes as follows: RUL 1.20 L (IQR 1.08–1.33 L), LUL 1.20 L (IQR 1.06–1.35 L), LM 0.45 L (IQR 0.39–0.57 L), RLL 1.42 L (IQR 1.11–1.58 L), and LLL 1.32 L (IQR 1.12–1.51 L). Median %LAAs at baseline CT was 0% (IQR 0–2.5%).

The median ASC ppo-FEV1 (1.81 L, IQR 1.43–2.35 L) was significantly (*p* < 0.0001) higher than WAL ppo-FEV1 (1.64 L, IQR 1.43–2.35 L). Both median ASC ppo-FEV1 (*p* = 0.006) and WAL ppo-FEV1 (*p* < 0.0001) were significantly lower than the measured post-operative FEV1. Similarly, ASC ppo-%DLCO (56%, IQR 48–68%) was significantly (*p* < 0.0001) higher than WAL ppo-DLCO% (54%, IQR 45–65%). Both ASC ppo-%DLCO (*p* = 0.046) and WAL ppo-%DLCO were significantly (*p* = 0.001) lower than the measured postoperative-%DLCO.

### 3.3. Correlation and Agreement Analysis

**Figure 2:**
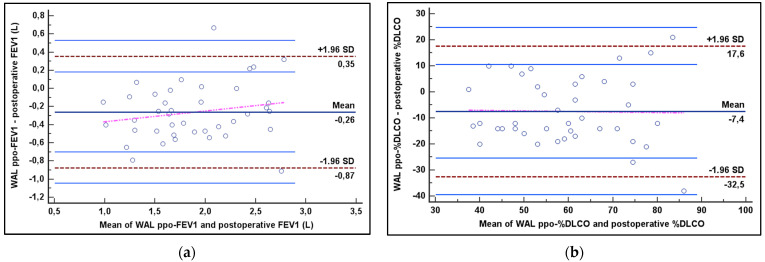
Agreement between ppo-WAL with both postoperative FEV1 (**a**) and %DLCO (**b**) according to the Bland–Altman plot. %DLCO = percentage of diffusing capacity of the lung for carbon monoxide; FEV1 = forced expiratory volume in 1 s; SD = standard deviation; and WAL = well-aerated lung.

**Figure 3:**
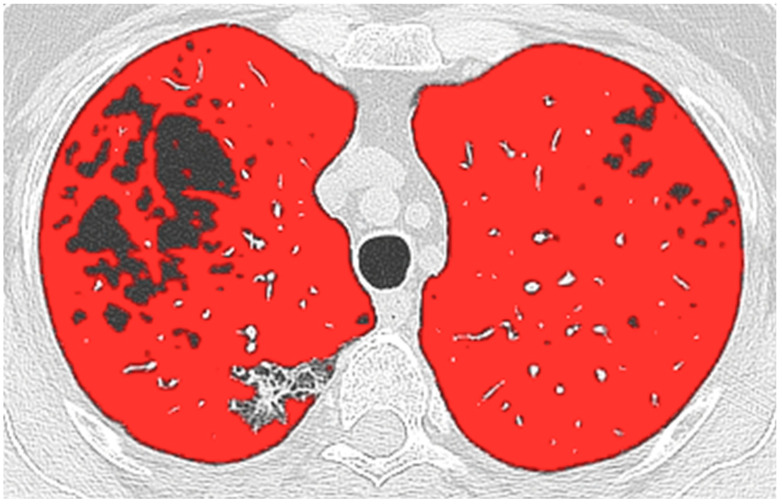
A 58 years-old female former smoker (30 pack-years) who underwent right upper lobe (RUL) lobectomy for adenocarcinoma, with 7% of emphysema on RUL (<−950 HU; black areas). The predicted postoperative forced expiratory volume in 1 s (FEV1) based on well-aerated lung (between −950 and −750 HU, WAL; highlighted in red) was 1.81 L, while with the anatomical segment counting (ASC) it was 2.08 L. At 12-months follow-up after lobectomy, the measured FEV1 was 1.71 L.

In Table 2 are summarized the findings of the correlation and agreement analysis. Postoperative FEV1 and %DLCO were significantly (*p* < 0.001) correlated with WAL ppo values (for FEV1 rho = 0.842; for %DLCO rho = 0.717). The agreement between WAL ppo-FEV1 and post-operative FEV1 was excellent, with an ICC of 0.904 (95% CI 0.819–0.949) and LoA included between +0.35 L and −0.87 L at Bland–Altman plot (Figure 2a and Figure 3). The agreement was good between post-operative %DLCO and WAL ppo-%DLCO, with an ICC of 0.770 (95% CI 0.566–0.878) and LoA ranging from +17% to −32% according to the Bland–Altman plot (Figure 2b and Figure 3).

## 4. Discussion

In our study, we demonstrated, in a cohort of 40 patients who underwent surgical lobectomy or segmentectomy for NSCLC, a significant correlation between WAL ppo-PFTs and postoperative PFTs measured 12 months after surgery [17]. In addition, the agreement between WAL ppo-PFTs with corresponding postoperative measured PFTs was excellent for FEV1 and good for %DLCO.

Well-ventilated regions of the lung may be a surrogate of respiratory function [18]. In patients affected by acute respiratory distress syndrome (ARDS), a ratio of less than 40% between the WAL region and whole-lung volume noted at CT was associated with a higher risk of death [18]. Furthermore, in patients affected by COVID-19 pneumonia, the WAL extent lower than near 70% of the whole-lung volume was related to an increased risk of death or intensive care unit admission [11,19]. We considered WAL the lung volume to include between −950 and −750 HU since it demonstrated better correlation with the visual score; in addition, pixels greater than −699 HU seem to represent lung parenchyma with no or reduced participation in gas exchange [20].

The WAL-estimated FEV1 and %DLCO values were significantly lower than the corresponding postoperative values, as previously reported [9]. This finding may be due to the different positions of the patients during CT examination and spirometry execution. At the supine position during CT, the dorsal portions of the lung are less ventilated, consequently with higher density, and are excluded from the WAL calculation. Nevertheless, the underestimation of CT can be regarded as a ‘‘margin of safety’’ from postoperative respiratory insufficiency.

We found a significant correlation between WAL ppo-PFTs and measured postoperative PFTs 12 months after surgery. Similar results was demonstrated by Sverzellati et al., who reported a significant correlation between estimated FEV1 and measured FEV1 values (r = 0.97) three months after surgery [9]. According with our findings, Yokoba et al. identified a significant correlation between postoperative FEV1 and %DLCO and corresponding predicted values obtained by QCT; nevertheless, our results showed a higher tendency to underestimate PFTs, likely for the narrower range of CT density considered as WAL in our study (between −950 and −750 HU vs. −950 and −600 HU) [8].

Other techniques can evaluate postoperative lung function, such as scintigraphy, CT ventilation imaging, or magnetic resonance imaging (MRI). In practice, scintigraphy is not widely employed in assessing patients for lobectomy because of the difficulty in interpreting the contribution of individual lobes to the overall ventilation or perfusion [6]. CT ventilation imaging may be a useful alternative method in preoperative evaluation for lung cancer patients; nevertheless, it requires double CT acquisition images at end-expiration and end-inhalation, increasing radiation exposure [7]. MRI allows for a noninvasive approach that is probably even more precise than QCT evaluation; however, it adds an expensive examination not widely available to the flow chart of the lung cancer evaluation [21]. The use of QCT prior to surgery has been advocated by the ERS\ESTS guidelines to estimate postoperative lung function [6]. Postoperative lung function assessment based on WAL evaluation could be considered an alternative tool among all of the techniques available, with reliable agreement with postoperative PFTs values. WAL does not require additional examination since it can be extracted from the staging CT, which is a standard step of the diagnostic-therapeutic process of patients affected by NSCLC [1]. Additionally, it is easily calculated even with open-source software and can estimate the contribution to lung function of the specific lobe that requires resection [11].

This study has several limitations. First, it is a retrospective study from a single hospital with a small number of patients; nevertheless, we selected patients without previous lung surgery, RT, ChT, or postoperative complications, with available PFTs 12 months after lung resection, in order to assess the reliability of the predicted PFTs based on WAL at staging CT. Second, WAL estimation can be affected by patients inspiration; nonetheless, Best et al. previously suggested that spirometric standardization might not be necessary for routine CT volume assessment [22]. Third, CT scans were performed with different scanner and reconstruction parameters; however, the reliable agreement identified between WAL predicted PFTs shows that this method can be applied with dissimilar CT scanner and technical details.

## 5. Conclusions

In conclusion, the estimated postoperative FEV1 and %DLCO obtained on the basis of WAL calculated at staging CT showed a significant correlation and reliable agreement with measured postoperative corresponding PFTs 12 months after surgery for NSCLC in a cohort of COPD and non-COPD patients. Since staging CT is a routine procedure in preoperative lung cancer evaluation, it may simply and reliably allow for a ‘‘one-stop shop’’ approach by providing morphologic and functional data. WAL can be easily calculated at CT with commercial and open-source software on the basis of a density mask even without the whole lung segmentation. Additionally, in patients with borderline lung function, predicted PFTs on the basis of WAL can estimate the real contribution of the lung portion that will be resected on the whole pulmonary function.

## Figures and Tables

**Table 1:** Patient and lung cancer characteristics with surgical details.

Variable	All Patients (*n* = 40)
Age (y)	68 (62; 74)
Gender	
males	26/40 (65%)
females	14/40 (35%)
Smoking history	
never	5/40 (12%)
former	26/40 (65%)
current	9/40 (23%)
COPD	25/40 (62%)
Tumor stage (TNM 8th edition)	
IA1	16/40 (40%)
IA2	8/40 (20%)
IA3	3/40 (8%)
IB	9/40 (22%)
IIB	3/40 (8%)
IIIA	1/40 (2%)
Histologic type	
adenocarcinoma	38/40 (95%)
squamous cell carcinoma	2/40 (5%)
Surgical resection	
right upper lobe	18/40 (45%)
left upper lobe	11/40 (27%)
right lower lobe	5/40 (12%)
left lower lobe	6/40 (16%)
Surgery type	
lobectomy	31/40 (78%)
typical segmentectomy	5/40 (12%)
atypical segmentectomy	4/40 (10%)

Data are shown as the median or number of patients with the interquartile range or percentage, respectively, in brackets. COPD = chronic obstructive pulmonary disease.

**Table 2:** Correlation and agreement analysis.

Comparison	Spearman rho Coefficient (*p* Value)	Limit of Agreement +1.96SD, −1.96 SD	ICC (95% CI)
WAL ppo-FEV1 (L) vs. ASC ppo-FEV1 (L) WAL ppo-FEV1 (L) vs. postoperative-FEV1 (L) ASC ppo-FEV1 (L) vs. postoperative-FEV1 (L)	0.957 (*p* < 0.001) 0.842 (*p* < 0.001) 0.856 (*p* < 0.001)	+0.20, −0.46 +0.35, −0.87 +0.47, −0.72	0.978 (0.958–0.988) 0.904 (0.819–0.949) 0.916 (0.841–0.955)
WAL ppo-%DLCO vs. ASC ppo-%DLCO WAL ppo-%DLCO vs. postoperative-%DLCO ASC ppo-%DLCO vs. postoperative-%DLCO	0.948 (*p* < 0.001) 0.717 (*p* < 0.001) 0.751 (*p* < 0.001)	+4, −12 +17, −32 +19, −26	0.977 (0.957–0.988) 0.770 (0.566–0.878) 0.796 (0.615–0.892)

ASC = anatomical segment counting; CI = confidence interval; %DLCO = percentage of diffusing capacity of the lung for carbon monoxide; FEV1 = forced expiratory volume in 1 s; ICC = intraclass correlation coefficient; ppo = predicted postoperative; SD = standard deviation; and WAL = well-aerated lung.

## Data Availability

The data presented in this study are available on request from the corresponding author. The data are not publicly available due to privacy policy restrictions.

## References

[1] Postmus P.E., Kerr K.M., Oudkerk M., Senan S., Waller D.A., Vansteenkiste J., Escriu C., Peters S. (2017). Early and Locally Advanced Non-Small-Cell Lung Cancer (NSCLC): ESMO Clinical Practice Guidelines for Diagnosis, Treatment and Follow-Up. Ann. Oncol..

[2] Bade B.C., Dela Cruz C.S. (2020). Lung Cancer 2020: Epidemiology, Etiology, and Prevention. Clin. Chest Med..

[3] Baratella E., Cernic S., Minelli P., Furlan G., Crim F., Rocco S., Ruaro B., Cova M.A. (2022). Accuracy of CT-Guided Core-Needle Biopsy in Diagnosis of Thoracic Lesions Suspicious for Primitive Malignancy of the Lung: A Five-Year Retrospective Analysis. Tomography.

[4] Baratella E., Fiorese I., Minelli P., Veiluva A., Marrocchio C., Ruaro B., Cova M.A. (2022). Aging-Related Findings of the Respiratory System in Chest Imaging: Pearls and Pitfalls. Curr. Radiol. Rep..

[5] Zhang H., Tian S., Wang S., Liu S., Liao M. (2022). CT-Guided Percutaneous Core Needle Biopsy in Typing and Subtyping Lung Cancer: A Comparison to Surgery. Technol. Cancer Res. Treat..

[6] Brunelli A., Charloux A., Bolliger C.T., Rocco G., Sculier J.P., Varela G., Licker M., Ferguson M.K., Faivre-Finn C., Huber R.M. (2009). ERS/ESTS Clinical Guidelines on Fitness for Radical Therapy in Lung Cancer Patients (Surgery and Chemo-Radiotherapy). Eur. Respir. J..

[7] Eslick E.M., Bailey D.L., Harris B., Kipritidis J., Stevens M., Li B.T., Bailey E., Gradinscak D., Pollock S., Htun C. (2016). Measurement of Preoperative Lobar Lung Function with Computed Tomography Ventilation Imaging: Progress towards Rapid Stratification of Lung Cancer Lobectomy Patients with Abnormal Lung Function. Eur. J. Cardio-Thorac. Surg..

[8] Yokoba M., Ichikawa T., Harada S., Shiomi K., Mikubo M., Ono M., Sonoda D., Satoh Y., Hanawa H., Naoki K. (2020). Comparison between Quantitative Computed Tomography, Scintigraphy, and Anatomical Methods for Prediction of Postoperative FEV1 and DLCO: Effects of Chronic Obstructive Pulmonary Disease Status and Resected Lobes. J. Thorac. Dis..

[9] Sverzellati N., Chetta A., Calabrò E., Carbognani P., Internullo E., Olivieri D., Zompatori M. (2005). Reliability of Quantitative Computed Tomography to Predict Postoperative Lung Function in Patients with Chronic Obstructive Pulmonary Disease Having a Lobectomy. J. Comput. Assist. Tomogr..

[10] Risoli C., Nicol M., Colombi D., Moia M., Rapacioli F., Anselmi P., Michieletti E., Ambrosini R., Di Terlizzi M., Grazioli L. (2022). Different Lung Parenchyma Quantification Using Dissimilar Segmentation Software: A Multi-Center Study for COVID-19 Patients. Diagnostics.

[11] Colombi D., Bodini F.C., Petrini M., Maffi G., Morelli N., Milanese G., Silva M., Sverzellati N., Michieletti E. (2020). Well-Aerated Lung on Admitting Chest CT to Predict Adverse Outcome in COVID-19 Pneumonia. Radiology.

[12] Goldstraw P., Chansky K., Crowley J., Rami-Porta R., Asamura H., Eberhardt W.E.E., Nicholson A.G., Groome P., Mitchell A., Bolejack V. (2016). The IASLC Lung Cancer Staging Project: Proposals for Revision of the TNM Stage Groupings in the Forthcoming (Eighth) Edition of the TNM Classification for Lung Cancer. J. Thorac. Oncol..

[13] Park K.J., Bergin C.J., Clausen J.L. (1999). Quantitation of Emphysema with Three-Dimensional CT Densitometry: Comparison with Two-Dimensional Analysis, Visual Emphysema Scores, and Pulmonary Function Test Results. Radiology.

[14] Howington J.A., Blum M.G., Chang A.C., Balekian A.A., Murthy S.C. (2013). Treatment of Stage I and II Non-Small Cell Lung Cancer: Diagnosis and Management of Lung Cancer, 3rd Ed: American College of Chest Physicians Evidence-Based Clinical Practice Guidelines. Chest.

[15] Zeiher B.G., Gross T.J., Kern J.A., Lanza L.A., Peterson M.W. (1995). Predicting Postoperative Pulmonary Function in Patients Undergoing Lung Resection. Chest.

[16] Schober P., Mascha E.J., Vetter T.R. (2021). Statistics From A (Agreement) To. Anesth. Analg..

[17] Colombi D., Di Lauro E., Silva M., Manna C., Rossi C., De Filippo M., Zompatori M., Ruffini L., Sverzellati N. (2013). Non-Small Cell Lung Cancer after Surgery and Chemoradiotherapy: Follow-up and Response Assessment. Diagn. Interv. Radiol..

[18] Nishiyama A., Kawata N., Yokota H., Sugiura T., Matsumura Y., Higashide T., Horikoshi T., Oda S., Tatsumi K., Uno T. (2020). A Predictive Factor for Patients with Acute Respiratory Distress Syndrome: CT Lung Volumetry of the Well-Aerated Region as an Automated Method. Eur. J. Radiol..

[19] Leoni M.L.G., Lombardelli L., Colombi D., Bignami E.G., Pergolotti B., Repetti F., Villani M., Bellini V., Rossi T., Halasz G. (2021). Prediction of 28-Day Mortality in Critically Ill Patients with COVID-19: Development and Internal Validation of a Clinical Prediction Model. PLoS ONE.

[20] Rienmüller R.K., Behr J., Kalender W.A., Schätzl M., Altmann I., Merin M., Beinert T. (1991). Standardized Quantitative High Resolution CT in Lung Diseases. J. Comput. Assist. Tomogr..

[21] Ohno Y., Hatabu H., Higashino T., Takenaka D., Watanabe H., Nishimura Y., Yoshimura M., Sugimura K. (2004). Dynamic Perfusion MRI versus Perfusion Scintigraphy: Prediction of Postoperative Lung Function in Patients with Lung Cancer. AJR. Am. J. Roentgenol..

[22] Best A.C., Meng J., Lynch A.M., Bozic C.M., Miller D., Grunwald G.K., Lynch D.A. (2008). Idiopathic Pulmonary Fibrosis: Physiologic Tests, Quantitative CT Indexes, and CT Visual Scores as Predictors of Mortality. Radiology.

